# WHO’s Salt Substitution Guidelines for Population-Wide Impact: Act on Strong Evidence, Monitor for the Long Term

**DOI:** 10.5334/gh.1419

**Published:** 2025-03-26

**Authors:** J. Jaime Miranda

**Affiliations:** 1 Sydney School of Public Health, Faculty of Medicine and Health, The University of Sydney, Sydney, Australia; 2 CRONICAS Centro de Excelencia en Enfermedades Crónicas, Universidad Peruana Cayetano Heredia, Lima, Peru

**Keywords:** Salt substitution, Sodium reduction, Potassium-enriched salt, Hypertension prevention, cardiovascular disease, Public health policy, WHO guidelines, Population-wide intervention, Food system reform, Health equity, Sustainable development, Regulatory strategies, Blood pressure control, Nutrition policy, Global health

Access to safe, nutritious, and adequate food is a fundamental human right, enshrined in the Universal Declaration of Human Rights (1). Yet, the reality for billions worldwide falls short of this standard, especially in low- and middle-income countries, where poor dietary quality fuels soaring rates of hypertension and cardiovascular disease (2,3,4). Excessive sodium intake remains one of the most significant yet preventable dietary risks, disproportionately affecting populations with limited access to healthcare and antihypertensive treatment (5,6). Recognising this, the World Health Organization (WHO) has issued a pivotal guideline endorsing salt substitutes to reduce hypertension and cardiovascular disease risk at the population level (7). Lower-sodium salt substitutes, where sodium chloride is partially replaced with potassium chloride, offer a practical, scalable way to reduce sodium intake while increasing potassium, a dual benefit for lowering blood pressure. However, their success depends on systemic implementation rather than individual dietary change. Governments must integrate salt substitution into national health strategies, public procurement policies, and industry regulations to achieve meaningful impact, making lower-sodium alternatives widely available and accessible.

## From Clinics to Communities: A Tipping Point for Salt Substitutes

Cardiovascular disease prevention has primarily focused on high-risk individuals, overlooking broader societal and environmental determinants of disease. This approach, often rooted in clinical cardiology and epidemiology, prioritises treating individual cases rather than addressing the causes of incidence at a population level. As Geoffrey Rose argued in ‘Sick Individuals and Sick Populations’ (8,9), the majority of disease cases do not arise from high-risk individuals but from the vast number of people at moderate risk, who collectively contribute most to the burden of disease.

Salt substitution exemplifies a population-wide prevention strategy. Instead of relying on individuals to change behaviour, it modifies the food environment, making healthier choices the default. This shift reduces hypertension and cardiovascular disease at scale, surpassing interventions focused solely on high-risk individuals. This shift recognises that high sodium intake is shaped by food systems, availability, and industry practices—factors requiring structural interventions, not just clinical management (10). While cardiology remains one of the most research-intensive fields in medicine, greater emphasis on pragmatic, system-wide interventions is needed to turn evidence into meaningful population-level change (11). High sodium intake is not an individual decision in isolation; it is a consequence of food systems where affordability, availability, and industry-driven product formulations limit healthier choices.

## Salt Substitutes in Policy: Moving Beyond Guidelines

The new WHO guideline endorses replacing regular salt (100% sodium chloride) with potassium-enriched salt substitutes as a proven strategy to lower blood pressure and reduce the burden of hypertension and cardiovascular disease (6). This recommendation builds on a growing body of evidence and policy momentum that has been years in the making. The World Heart Federation’s Hypertension Roadmap (12), the European Society of Cardiology Hypertension Guidelines (13), and the Brazilian Hypertension Guidelines (14) all support salt substitution as an essential intervention.

Regulatory action is aligning with clinical guidelines. The US Food and Drug Administration (FDA) is assessing the expansion of salt substitutes in food manufacturing, a move that could set a global precedent (15,16,17). The question is no longer whether salt substitution should be implemented, but rather how rapidly and equitably governments and industry can scale it up to maximise public health impact.

## A Simple, Scalable Solution for Prevention

Salt substitutes offer a practical and scalable solution for sodium reduction, particularly in low- and middle-income countries. Unlike interventions requiring major behavioural shifts, such as reducing discretionary salt use, salt substitution fits into existing habits, offering a simple swap rather than a complete dietary overhaul (18). This distinction is crucial, as public health interventions that align with existing consumption patterns tend to achieve higher adherence and impact at scale. In China, a Iarge-scale trial found that substituting regular salt with 75% sodium chloride and 25% potassium chloride led to a 14% reduction in stroke risk and a 12% decline in cardiovascular mortality (19). Similarly, in a stepped-wedge cluster-randomised trial in rural Peru, introducing a potassium-enriched salt substitute resulted in reductions in systolic and diastolic blood pressure and a 50% reduction in new hypertension cases over three years (18). These findings highlight salt substitutes as a high-impact strategy for reducing cardiovascular disease risk at a population level, especially in regions where medical treatment for hypertension remains out of reach for many.

## Beyond Health: Salt Substitution as a Driver of Sustainable Development

The most recent Sustainable Development Goals (SDGs) report states that ‘transformations are inevitable’, emphasising the need for strategies that maximise interlinkages and synergies across SDGs to accelerate progress towards sustainable development (20). Salt substitution exemplifies this integrated approach, extending beyond the expected links to nutrition (SDG 2), health (SDG 3), and inequality reduction (SDG 10) to also support multiple SDGs. By lowering dietary sodium, salt substitution enhances workforce productivity and reduces healthcare costs (SDG 8, Decent Work and Economic Growth). It also supports SDG 9 (Industry, Innovation, and Infrastructure) by improving access to an affordable, health-improving alternative to regular salt, which requires policy support for market penetration. Salt substitution also intersects with SDG 13 (Climate Action) by reducing dependence on environmentally damaging salt mining and promoting more sustainable food production (21,22). Critically, SDG 17 (Partnerships for the Goals) is essential to its success. Achieving widespread adoption requires international cooperation (Target 17.6) to share research and best practices and public-private partnerships (Target 17.7) to scale implementation without exacerbating health inequities. Salt substitution represents a systems-level solution, integrating public health, economic sustainability, and environmental resilience.

## A Public Health Breakthrough: Salt Substitution as a Game-Changer

Salt substitution is not merely another dietary recommendation; it is a game-changer, much like antiretrovirals for HIV, statins for cardiovascular prevention, and semaglutide for obesity and diabetes management. Each of these interventions was implemented before long-term data were complete, with the understanding that delaying adoption would have cost millions of lives. Public health history shows that major breakthroughs often face hesitation at first. Universal salt iodisation, now recognised as one of the most successful global nutrition policies of the 20th century, once faced similar doubts before virtually eliminating iodine deficiency disorders worldwide. The lesson is clear: waiting for decades of additional research before implementing population-wide salt substitution is neither practical nor ethical. As with vaccines, trans fat bans, and iodisation, surveillance must happen in parallel with implementation, not before it.

Resistance is inevitable, as seen in past public health reforms that challenged industry norms, from the elimination of trans fats to taxation on sugary beverages. Every major shift in food policy has faced opposition, yet these measures have proven critical in reducing diet-related diseases. As El Quijote reminds us: *‘Ladran, Sancho, señal que avanzamos’* (They bark, Sancho, a sign we are moving forward) (23), global health institutions, national regulators, and policymakers embracing salt substitution signal a major shift in the field.

## Regulation Without Exclusion: Ensuring Equitable Implementation

Despite the clear benefits, scaling up salt substitution at a global level presents logistical and regulatory challenges. Availability, affordability, and food industry reformulation remain major hurdles. Regular salt is a cheap, essential food ingredient; altering its formulation has economic and trade implications. If lower-sodium alternatives are mandated without price controls or subsidies, manufacturers may pass costs onto consumers, exacerbating dietary inequities. Food companies may also resist reformulation due to concerns about production costs, supply chains, and consumer acceptance. The policy response must therefore balance public health goals with industry incentives, using a combination of regulation, taxation, and subsidies to ensure widespread adoption without widening health disparities.

## Monitoring for Progress, Not Delay

Long-term success requires ongoing surveillance, similar to vaccine pharmacovigilance. While salt substitutes reduce hypertension and CVD, concerns about hyperkalaemia warrant monitoring. However, large trials, including in elderly care, show minimal risk (24,25). Post-market surveillance should facilitate progress, not stall it. Just as vaccines and many other life-saving drugs were introduced with built-in safety monitoring, salt substitution policies must include real-world surveillance to refine and scale implementation without unnecessary delays.

## Beyond Personal Choice: A Systemic Shift for Health and Equity

The next step is action, but also recognition of a fundamental transformation. For decades, public health messaging has urged individuals to ‘eat less salt’ with limited success. WHO’s endorsement signals a paradigm shift—making sodium reduction achievable without radical behaviour change. This is a rights-based approach, shifting responsibility from individuals to systemic solutions. Endorsement by major professional bodies signals growing global consensus that salt substitution is not an experiment; it is an essential intervention. This is more than a medical intervention for individual patients; it is a societal-level transformation, enabling governments to implement an equity-driven strategy, making cardiovascular prevention more pragmatic, scalable, and accessible. Scaling up implementation through coordinated policies and cross-sector collaboration is essential to ensure salt substitution achieves not just cardiovascular benefits but broader societal transformation in line with the SDG agenda. The shift from individual responsibility to systemic action is long overdue. Salt substitution offers a pragmatic, scalable, and rights-based solution that can transform cardiovascular prevention. The opportunity is clear, the evidence is strong, and governments must act now to ensure this breakthrough reaches the populations that need it most.
